# What can we do about myopia? An evidence-informed approach

**Published:** 2024-05-15

**Authors:** Padmaja Sankaridurg, Huy Tran, Nina Tahhan, Jude Stern

**Affiliations:** 1 Conjoint Professor: School of Optometry and Vision Science, University of New South Wales, Sydney, Australia and Head, Global Myopia Management: ZEISS VisionCare, Aalen, Germany.; 2 Lecturer: Department of Ophthalmology, University of Medicine and Pharmacy at, Ho Chi Minh City, Vietnam.; 3 Executive Director: International Myopia Institute, Sydney, Australia.; 4 Head of Knowledge Management: International Agency for the Prevention of Blindness, Sydney, Australia.

**Prevention is vital to reduce the number of people with myopia, and management strategies are effective in reducing the progression of myopia and decreasing the risk of sight-threatening complications.**

**Figure:**
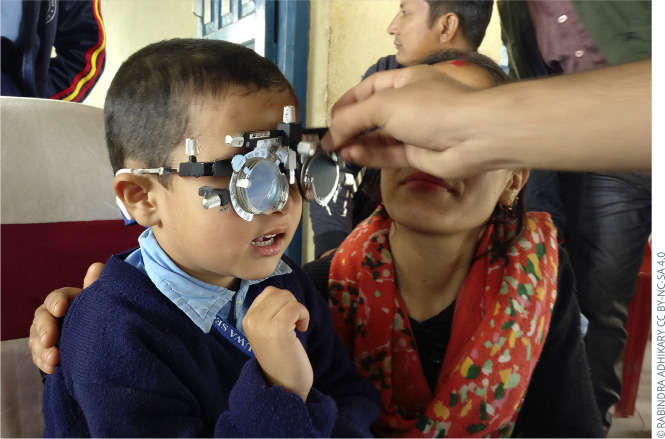
By 2050, it is estimated that half the world's population will have myopia. nepal

Myopia – short-sightedness – is a significant problem around the world. Currently, one in three people on the planet have myopia; by the year 2050, it is estimated that one in every two individuals will be affected.

Myopia typically starts to develop in childhood and tends to progress to higher levels until adulthood. The earlier myopia starts, the more likely a child will end up with high levels of myopia, which puts them at greater risk of complications, such as myopic maculopathy, which may result in loss of vision. Whilst any level of myopia may cause complications, the risk increases with increasing levels of myopia.^1^

“Myopia typically starts to develop in childhood and tends to progress to higher levels until adulthood.”

## Who is at risk?

Those who are most at risk of developing myopia are children who spend less time outdoors and more time indoors on near tasks, children who have higher levels of education, and children who have parents with myopia. People of East Asian ethnicity are also at higher risk.

## Impact of myopia

If left uncorrected, poor vision in childhood can hinder school performance and wellbeing. Quality of life is affected in people with higher levels of myopia and people with myopia-related complications. Given the progressive nature of the condition, myopia has a financial impact on the families of affected individuals and on society, due to the health expenditure related to the costs of managing the condition as well as costs related to eye examinations, corrective devices, and lost productivity.

The rising prevalence of myopia is expected to strain eye care, health, and education systems. Vision loss due to myopia may result in productivity loss and the resulting economic burden may be significant for those countries with a higher prevalence of myopia.

## Prevention

Due to the progressive nature of myopia, preventing or delaying the onset is currently the most effective way to tackle the burden of myopia .

It is well established that increasing time outdoors can greatly reduce the risk of developing myopia. Therefore, the current recommendation is that **children should spend at least 2 hours outside each day** to prevent or delay the onset of myopia.^2^

Public messages about the prevention of myopia is the responsibility of all stakeholders, including researchers, clinicians, health bodies, and governmental authorities. To achieve widespread public awareness, various channels of communication such as engaging with parents and carers, involvement in community groups and schools, as well as print and social media campaigns should be considered.

## Treatment

Once myopia has been diagnosed, it is possible to offer treatment that not only corrects visual symptoms, but also slows progression. (Note: a comprehensive eye examination for children should include cycloplegia if possible; see the article on cycloplegia in this issue).

There are a growing number of management options that have been shown to be effective at slowing the progression of the condition, including:

- Specially designed spectacle lenses and contact lenses
- Orthokeratology
- Eye drops containing low concentrations of atropine
- Light-based therapies
- Combination treatments, involving one or more of these categories.

Research into the efficacy of different myopia control interventions is ongoing,^3^ which makes it challenging to stay up to date with the latest scientific evidence.

Several organisations and professional bodies have developed resources to help clinicians make evidence-informed decisions about myopia control for their patients.

The IMI's reviews of evidence, published as open access white papers (tinyurl.com/IMI-whitepapers) have been summarised in a 2-page infographic, available at myopiainstitute.org/myopia-infographics/

The World Council of Optometry (WCO) has developed a ‘Standards of Care’ guideline (bit.ly/WCOstandard) and a practical guide to managing children with myopia (bit.ly/4agfYxu).

For clinical guidelines, it may be useful to refer to optometry and ophthalmology associations. They have an obligation to stay up to date and their purpose is to help clinicians – look to your local associations for relevant local guidelines. They do vary, and some suggest referral pathways. In addition to clinical efficacy, there are several other factors that will guide the choice, implementation and success of myopia management options, including:

- The availability of different interventions in your country, and whether they have been approved by professional and regulatory bodies.
- The cost of interventions, and patients’ ability to pay for them.
- How acceptable the interventions are to patients. This could depend on comfort and visual performance, for example.
- How familiar practitioners are with the different myopia control strategies, and how confident they feel about implementing them.

### Are these treatments safe and comfortable?

Overall, adverse events associated with various myopia control strategies have been reported to be minimal, although more data is needed to establish the safety of light therapy.

Using atropine at concentrations higher than 0.01% may cause side effects such as increased pupil size, accommodation paralysis, photophobia, glare, and blurred near vision, but these effects are reversible when treatment is stopped.

“Prevention of myopia through lifestyle changes is still the most effective way to address this growing problem.”

Due to the optical design features needed for spectacles and contact lenses used in myopia control, visual disturbances have been reported, which vary with lens design. These include haze and ghosting (with multifocal contact lenses) and reduced visual performance in the periphery (with certain spectacle lens designs). The latter is usually limited to one line reduction in the peripheral treatment areas of the spectacle lenses.

Although complications with contact lenses and orthokeratology are rare, they include microbial keratitis and infiltrates, with daily disposable wear minimising such risks.

The safety of low-level red-light therapy requires further study, although known side effects involve temporary after-flash images and rare cases of retinal damage that resolved without lasting effects.

**How is the effectiveness of myopia control strategies measured?**

The effectiveness of interventions to slow myopia progression can be measured by observing the change in refractive error, measured in dioptres, or the change in axial length, measured in mm.

Effectiveness is often reported as ‘relative efficacy’. In these studies, the progression of refractive error (in dioptres) in the group using the myopia intervention is compared with the progression in a control group of children using standard single-vision spectacles or contact lenses. Because control groups can differ between studies, it is important to be cautious when using this measurement to compare different treatment options.

Another approach to measuring the effectiveness of interventions is to compare myopia control strategies to the natural eye growth observed in non-myopic (emmetropic) eyes. Axial length measurements are also considered more accurate than refractive error measurements for assessing progression.

### A note on rebound

With myopia control treatments involving higher doses of atropine and red-light therapy, an accelerated increase in myopia has been reported once these treatments are stopped. This effect, known as rebound, tends to be more pronounced in initial months after treatment is stopped. To mitigate rebound, a tapered approach is recommended, although the efficiency of tapering methods needs further evaluation. With optical strategies, studies with orthokeratology indicate some rebound when switching from orthokeratology to single vision lenses, but no rebound was reported with contact lenses or spectacles.

## Conclusion

In conclusion, prevention of myopia through lifestyle changes is still the most effective way to address this growing problem. Myopia management approaches have been growing and have proven to be effective in reducing the risk of myopia progression. Data and modelling indicate that various approaches have the potential to positively influence outcomes for individuals as well as benefit society at large. Ongoing research and advancement will continue to shape the future landscape for myopia prevention and management.

## References

[1] Sankaridurg P Tahhan N Kandel H. IMI Impact of Myopia. Invest Ophthalmol Vis Sci 2021;62(5):2. doi: 10.1167/iovs.62.5.2 10.1167/iovs.62.5.2 PMC8083082 33909036

[2] Morgan IG Wu PC Ostrin LA. IMI Risk Factors for Myopia. Invest Ophthalmol Vis Sci 2021;62(5):3. doi: 10.1167/iovs.62.5.3 10.1167/iovs.62.5.3 PMC8083079 33909035

[3] Sankaridurg P Berntsen DA Bullimore MA. IMI - 2023 Digest. Invest Ophthalmol Vis Sci 2023;In Press 10.1167/iovs.64.6.7 PMC10155872 37126356

